# Coping strategies and social support among caregivers of patients with cancer: a cross-sectional study in Vietnam

**DOI:** 10.3934/publichealth.2021001

**Published:** 2020-12-11

**Authors:** Nguyen Xuan Long, Nguyen Bao Ngoc, Tran Thi Phung, Dao Thi Dieu Linh, Ta Nhat Anh, Nguyen Viet Hung, Nguyen Thi Thang, Nguyen Thi Mai Lan, Vu Thu Trang, Nguyen Hiep Thuong, Nguyen Van Hieu, Hoang Van Minh

**Affiliations:** 1 University of Languages and International Studies, Vietnam National University, Hanoi, Vietnam; 2 Center for Population Health Sciences, Hanoi University of Public Health, Hanoi, Vietnam; 3 Graduate Academy of Social Sciences, Vietnam Academy of Social Sciences, Hanoi, Vietnam; 4 International School, Vietnam National University, Hanoi, Vietnam; 5 Hanoi National University of Education, Hanoi, Vietnam

**Keywords:** coping, social support, caregivers, cancer, Vietnam

## Abstract

Research on coping strategies and social support among Vietnamese cancer caregivers remains limited. In this study, we aim to examine the relationships between types of coping strategies utilized and social support among cancer caregivers. This was a cross-sectional study conducted in three main cancer hospitals in the Northern, Central and Southern regions of Vietnam. The 28-item Brief COPE Inventory (BCI) Scale and the Multidimensional Scale of Perceived Social Support (MSPSS) were utilized. Descriptive statistics and multivariate linear regression were performed. Active coping, acceptance and positive reframing were the most used coping strategies among participants, while substance use was the least commonly used. Level of social support was positively correlated with the utilization of coping mechanisms. Receiving high social support and utilizing positive coping strategies enables caregivers to mitigate their caregiving burden, control the situation and enhance their own quality of life.

## 1. Introduction

In recent years, the global burden of cancer has rapidly increased. Cancer is currently the second leading cause of death in the world, following only cardiovascular diseases [1]. Since cancer is a chronic disease, patients as well as their caregivers are forced to confront the long journey of cancer treatment and care, which may lead to various psychological and mental health issues [2]–[4]. Previous scientific evidence indicates that caregivers of patients diagnosed with cancer suffer from high levels of negative emotions and psychological distress including depression and anxiety [5]–[9]. A study by Geng and colleagues demonstrated that anxiety and depression are present in 46.5% and 42.3% of cancer caregivers respectively [5]. Further, Perez et al. found the rate of anxiety among these caregivers can reach as high as 76% [10]. It is conventional for cancer caregivers to use several coping strategies which may reduce and even eliminate the adverse impacts of anxiety-and-depression-creating problems; conversely, some coping strategies can also exacerbate these same issues [11].

By definition, “coping” refers to different ways or styles in which an individual may respond, behave and perform to deal with psychological distress or mental challenges [12],[13]. The coping process involves constant adaptations of people in their own circumstances [12],[14]. Previous findings show that caregivers of cancer patients in different context employ various coping strategies to confront the stressful events they encounter [15]–[21]. Among those coping styles, people seem to prefer problem-focused coping to emotion-focused coping [22]. Their ways of coping play a pivotal role in determining the influence of the stressor on their mental health status, when considering it in the context of culture, society and environment [23]. In addition, applying appropriate and effective coping strategies enables cancer caregivers to mitigate their caregiving burden, reduce psychological pressure and thereby improve their quality of life [11],[24],[25].

There have been various ways to define social support, which is often recommended to be vital to enhancing mental health [26]. According to Roohafza et al., “social support refers to the experience of being valued, respected, cared about, and loved by others who are present in one's life” [27]. It can be derived from distinct sources, such as friends, relatives, spouses, or the community. Social support may help to bolster individuals' self-esteem or provide them with assistance in relieving stress [28].

A literature review indicated a correlation between social support and coping strategies. Roohafza and colleagues found that perceived friend support has a significant positive correlation with an active coping style but a significant negative correlation with avoidant coping style [27]. It has also been found that social support can increase proactive coping [27],[29],[30]. Some previous studies suggest that “the relationship between styles of coping and social support should be considered as reciprocal rather than causal” [31],[32]. A study among family caregivers of patients diagnosed with esophageal cancer in China revealed significant associations between social support and emotion-focused coping, adaptive coping, and maladaptive coping styles [33].

Vietnam is a lower-middle-income country located in Southeast Asia. In 2018, Vietnam had an age-standardized cancer incidence rate of 151.4 per 100,000 people, corresponding to 164,671 new cancer cases [34]. In Vietnam, ways of coping and the utilization of social support may be influenced by cultural norms, interpersonal relationships, and family responsibilities. Examining ways of coping among cancer caregivers can help to strengthen positive coping strategies and reduce negative coping strategies. However, in Vietnam, research on caregivers of patients with cancer, especially about their coping styles with mental health challenges remains limited. There were only two studies by Nguyen Thi Thanh Mai and her colleagues in 2012 and 2013 on how parents of children with cancer cope with caregiver burden found parents have different ways of coping with negative emotions in throughout their children's different cancer stages, and also suggested the need to have psychosocial support for parents of children with cancer [35],[36]. Furthermore, to the best of our knowledge, none of the previous research in Vietnam considers the association between social support and coping strategies among caregivers of patients with cancer. Accordingly, in this study, we aim to identify the coping strategies utilized by the caregivers of Vietnamese cancer patients in controlling their psychological distress and evaluate the relationship between the coping strategies and social support among the Vietnamese caregivers. The evidence gained from this study is a crucial foundation for the development of appropriate interventions to support these people in the future.

## 2. Materials and methods

### 2.1. Study design

This research was conducted using a cross-sectional design.

### 2.2. Study participants

The participants of this study were the caregivers of Vietnamese cancer patients, responsible for attending to the needs of cancer patients. They were family members or distant relatives, providing unpaid, nonprofessional care and met the following criteria: (a) a primary care provider of a cancer patient who was diagnosed by a clinicianp; (b) ≥18 years old; and (c) able to participate in the study both physically and mentally.

### 2.3. Study setting

The study was conducted in three cancer hospitals located in three major cities in Vietnam: Vietnam National Cancer Hospital (located in Tan Trieu, Hanoi), Da Nang Oncology Hospital (Da Nang city) and Ho Chi Minh Oncology Hospital (Ho Chi Minh city). These such hospitals are the main cancer care centers in the Northern, Central and Southern regions of Vietnam, respectively. In each hospital, three departments were selected as research sites, namely the Departments of Radiation Oncology, Abdominal Surgery, and Neurological Surgery.

### 2.4. Sample size and sampling

All caregivers of patients diagnosed with cancer who were treated at one of the three afore-mentioned departments from October 10th to October 25th, 2019 and met the inclusion criteria were invited to this study. 789 caregivers of cancer patients were invited to participate in the survey.

### 2.5. Measurement

#### 2.5.1. Dependent variables

Coping strategies among the caregivers of cancer patients were measured using the 28-item Brief COPE Inventory (BCI) [37]. The scale comprises of 14 domains (two items per domain) and is divided into three groups of coping: problem-focused coping (planning, active coping and use of instrumental support); emotion-focused coping (acceptance, positive reframing, use of emotional support, humor, religion) and dysfunctional coping (venting, denial, self-blame, self-distraction, substance use, behavioral disengagement) [37].

Participants were asked to rate each item on a 4-point Likert scale with 1 = “I haven't been doing this at all” and 4 = “I've been doing this a lot”. The BCI is a validated and frequently used tool for determining the ways people confront a problem [38],[39].

#### 2.5.2. Independent variables

The socioeconomic and demographic information collected from the caregivers included: sex (male/female), age (<45 years old/≥45 years old), education, occupation, relationship to the patient (spouse/child/others), type of support given to the patient, financial difficulty (yes/no), location (Ha Noi, Da Nang, Ho Chi Minh) and social support.

Level of education was grouped as (a) incomplete secondary school (did not complete the ninth grade or lower); (b) secondary school or higher (completion of the ninth grade or higher).

Occupational status of the caregivers was defined by two groups: (a) unemployed (person who is retired, studying or does not have any paying job); (b) employed (currently working and receiving income for the work).

Support to the patients was grouped into three categories: (a) primarily provide for both finance and care; (b) primarily provide for either finance or care; and (c) other (partially provide finance, care and/or different support).

Social support was identified using the Multidimensional Scale of Perceived Social Support (MSPSS) [40]. This questionnaire consists of 12 items to determine the different kinds of support from friends, family, and significant others. Each question can be responded to through a rating scale, ranging from 1 (very strongly disagree) to 7 (very strongly agree). Total MSPSS scores ranged from 12 to 84 and were classified into three groups: low support (12–47), moderate support (48–68), and high support (69–84) [33].

#### 2.5.3. Translation of the instruments

The MSPSS and Brief COPE questionnaire were translated into Vietnamese by a local professional translator, then another independent linguistic expert provided back-translation from those instruments into English. Finally, a third qualified translator compared both English versions to ensure uniformity of meaning.

### 2.6. Data collection

Data was collected via individual interviews with three interviewers in each hospital. The interviewers received an eight-hour training session to assure the quality and consistency of the data collected. Using the eligibility screening, the interviewers approached caretakers from each of the study sites during the study period to explain the aims of the study, request participation, and obtain informed consent. The interviewers also informed the participants that their participation was entirely voluntary, and they have the right to withdraw at any time.

### 2.7. Statistical methods

Both descriptive and analytical statistics were performed using Stata 14 software (Stata Corporation). Descriptive statistics of the scores for coping strategies among the study participants were calculated, including mean, median, minimum, maximum, and standard deviation. Bivariate linear regression was utilized to examine the association between the score given by the participant to each coping mechanism and the level of social support (MSPSS scores) among the study participants. Multivariate linear regression modelling was performed to evaluate the association between the score of each coping strategy domain and the level of social support among the caregivers while controlling for their sociodemographic status. A significance level of p < 0.05 was used.

## 3. Results

Of the 789 caregivers of cancer patients invited, 730 agreed to participate and 579 completed the survey (participation rate of 73.38%). The majority of caregivers were women (68.5%; n = 396), completed secondary or higher education (79.2%; n = 412) and married to the patients (46.8%; n = 270) (Table 1). Almost 58% of caregivers were 45 years of age or older. For employment status, 93.5% (n = 519) of caregivers reported that they had a paid job at the time of the interview. Almost half of the participants (48.1%; n = 275) were primarily responsible for both finances and care to the patient. The results show the majority of caregivers were under a financial burden (82.1%; n = 473). Levels of social support were relatively evenly distributed among the participants, with low, middle, and high social support being reported at 29.7% (n = 172), 37.1% (n = 215), and 33.2% (n = 192), respectively.

When asked about the ways caregivers confront the issue, the results, as defined by the BCI, showed that the caregivers' mean level of utilizing emotional strategy coping mechanisms was 31.4 ± 4.8, while the mean score of using the problem strategy and dysfunctional strategy were lower with 20.2 ± 3.2 and 29.6 ± 6.7, respectively.

Table 2 presents the mean scores and standard deviation of 14 coping styles among the study caregivers. The three leading coping strategies utilized were: acceptance (6.9 + 1.4), active coping (7.2 ± 1.3) and positive reframing (7.0 ± 1.3). Results demonstrated that substance use was the least utilized way to cope (3.3 ± 1.7).

**Table 1.** General characteristics of the study caregivers.

Factor	Value
N	579
Sex	
Men	182 (31.5%)
Women	396 (68.5%)
Caregivers age	
<45	247 (42.7%)
≥45	332 (57.3%)
Education	
Incomplete secondary school	153 (27.1%)
Secondary school and higher	412 (72.9%)
Occupation	
Unemployed	36 (6.5%)
Employed	519 (93.5%)
Relationship to patient	
Spouse	270 (46.8%)
Children	139 (24.1%)
Others	168 (29.1%)
Support given to patient	
Finance and Care	275 (48.1%)
Finance or Care	196 (34.3%)
Other	101 (17.7%)
Financial difficulty	
Yes	473 (82.1%)
No	103 (17.9%)
Location	
Hanoi	438 (75.6%)
Da Nang	63 (10.9%)
Ho Chi Minh	78 (13.5%)
Social Support (defined by MSPSS)	
Low support	172 (29.7%)
Moderate support	215 (37.1%)
High support	192 (33.2%)
Coping dimensions	
Problem focused Coping, mean (SD)	20.2 (3.2)
Emotion focused Coping, mean (SD)	31.4 (4.8)
Dysfunctional Coping, mean (SD)	29.6 (6.7)

**Table 2.** Mean score and standard deviation of coping strategies among cancer caregivers.

Factor	Mean (Standard deviation)
N	579
Active Coping	7.2 (1.3)
Planning	6.2 (1.6)
Instrumental Support	6.8 (1.4)
Emotional Support	6.7 (1.6)
Positive reframing	7.0 (1.3)
Acceptance	6.9 (1.4)
Religion	5.3 (2.2)
Humor	5.5 (1.5)
Self-Distraction	5.9 (1.8)
Venting	6.1 (1.5)
Denial	5.8 (1.9)
Behavioral Disengagement	4.1 (2.0)
Substance use	3.3 (1.7)
Self-Blame	4.4 (2.0)

All the different coping strategies were significantly associated with the level of support that caregivers reported receiving from social activities (p < 0.001), except three strategies (behavioral disengagement, substance use and self-blame). Overall, the participants who reported receiving high social support had higher mean scores in almost all the strategies as compared to those who reported low or moderate social support.

**Table 3.** Bivariate analysis of associations between caregivers' social support and different coping strategies.

Coping strategies	Social support			p-value
	Low support Mean (SD)	Moderate support Mean (SD)	High support Mean (SD)	
N	172	215	192	
Active Coping	6.7 (1.5)	7.3 (1.1)	7.5 (1.0)	<0.001
Planning	5.7 (1.7)	6.2 (1.7)	6.6 (1.5)	<0.001
Instrumental Support	6.1 (1.5)	7.0 (1.2)	7.2 (1.1)	<0.001
Emotional Support	6.0 (1.9)	6.7 (1.4)	7.2 (1.2)	<0.001
Positive reframing	6.5 (1.6)	7.2 (1.1)	7.3 (1.1)	<0.001
Acceptance	6.5 (1.7)	6.9 (1.3)	7.3 (1.1)	<0.001
Religion	4.8 (2.2)	5.2 (2.2)	5.8 (2.1)	<0.001
Humor	5.1 (1.4)	5.5 (1.4)	5.9 (1.6)	<0.001
Self-Distraction	5.3 (1.8)	6.0 (1.7)	6.3 (1.8)	<0.001
Venting	5.7 (1.6)	6.1 (1.5)	6.5 (1.3)	<0.001
Denial	5.3 (2.1)	5.8 (1.9)	6.2 (1.8)	<0.001
Behavioral disengagement	4.1 (1.9)	4.1 (2.0)	4.1 (2.1)	0.96
Substance use	3.5 (1.6)	3.3 (1.9)	3.1 (1.6)	0.068
Self-Blame	4.6 (1.9)	4.2 (1.8)	4.4 (2.2)	0.21

Table 4 illustrates the correlation between social support and three coping dimensions among the caregivers of cancer patients in our study. Results from simple linear regression analysis show that there were statistically significant associations between the level of social support and each coping dimensions. In particular, moderate and high support were both significantly positively correlated with both the problem-focused coping style (coefficient 1.93, [95% CI: 1.33, 2.54] and coefficient 2.78, [95% CI: 2.16, 3.40], respectively) and the emotion-focused coping style (coefficient 2.65, [95% CI: 1.76, 3.54] and coefficient 4.56, [95% CI: 3.65, 5.47], respectively). Regarding the dysfunctional coping styles, high support had a significant positive association (coefficient 2.15, [95% CI: 0.77, 3.53]), meanwhile, moderate support had no statistically significant correlation (coefficient 1.12, [95% CI: −0.22, 2.46]).

**Table 4.** Relationship between social support and three coping dimensions.

Social support (defined by MSPSS)	Coping dimensions					
	Problem_focused Coping Coefficient [95% CI]		Emotion_focused Coping Coefficient [95% CI]		Dysfunctional Coping Coefficient [95% CI]	
Low support	0.00	[0.00, 0.00]	0.00	[0.00, 0.00]	0.00	[0.00, 0.00]
Moderate support	1.93***	[1.33, 2.54]	2.65***	[1.76, 3.54]	1.12	[−0.22, 2.46]
High support	2.78***	[2.16, 3.40]	4.56***	[3.65, 5.47]	2.15**	[0.77, 3.53]
Constant	18.59***	[18.14, 19.04]	28.92***	[28.26–29.59]	28.50***	[27.5, 29.5]
r2	0.12		0.14		0.02
N	579.00		579.00		579.00

Note: *p < 0.05; ** p < 0.01; *** p < 0.001.

Three linear regression models were constructed, linking social support with each dimension of problem-focused, emotion-focused, and dysfunctional coping. Such models were controlled for caregiver's gender, age group, educational level, occupation, type of support given, and financial difficulty (Table 5). In these models, after adjusting for other general characteristics of study participants, higher social support level was significantly correlated with higher scores of problem-focused coping (coefficient 2.66, [95% CI: 2.00, 3.32]), emotion-focused coping (coefficient 4.39, [95% CI: 3.40, 5.37]) and dysfunctional coping styles (coefficient 2.13, [95% CI: 0.65, 3.61]). Moderate social support was also significantly associated with higher scores of problem-focused coping (coefficient 1.82, [95% CI: 1.19, 2.45]) and emotion-focused coping styles (coefficient 2.54, [95% CI: 1.59, 3.48]).

The results also showed that the 45 years and older age group was significantly negatively related to problem-focused coping style (coefficient −0.55, [95% CI: −1.08, −0.02]). Having no financial difficulty had a significant inverse correlation with problem-focused coping style (coefficient −0.80, [95% CI: −1.48, −0.12]) and dysfunctional coping style (coefficient −1.97, [95% CI: −3.49, −0.44]). Being employed was associated with dysfunctional coping style (coefficient 3.19, [95% CI: 0.71, 5.67]). Supporting the patient in either finance or care was significantly correlated to emotion-focused coping style (coefficient 1.14, [95% CI: 0.28, 2.00]) and dysfunctional coping style (coefficient 2.25, [95% CI: 0.96, 3.54]). Gender and education level were not found to be significantly correlated with any coping strategy.

**Table 5.** Linear regression analysis of factors associated with three coping dimensions.

	Coping dimensions					
	Problem_focused Coping Coefficient [95% CI]		Emotion_focused Coping Coefficient [95% CI]		Dysfunctional Coping Coefficient [95% CI]	
Social support (defined by MSPSS)
Low support	0.00	[0.00, 0.00]	0.00	[0.00, 0.00]	0.00	[0.00, 0.00]
Moderate support	1.82***	[1.19, 2.45]	2.54***	[1.59, 3.48]	0.89	[−0.53, 2.31]
High support	2.66***	[2.00, 3.32]	4.39***	[3.40, 5.37]	2.13**	[0.65, 3.61]
Gender						
Men	0.00	[0.00, 0.00]	0.00	[0.00, 0.00]	0.00	[0.00, 0.00]
Women	−0.09	[−0.64, 0.45]	0.77	[−0.04, 1.59]	−0.18	[−1.41, 1.04]
Caregivers age group
<45	0.00	[0.00, 0.00]	0.00	[0.00, 0.00]	0.00	[0.00, 0.00]
≥45	−0.55*	[−1.08, −0.02]	−0.48	[−1.27, 0.31]	−0.71	[−1.90, 0.48]
Education						
Incomplete secondary school	0.00	[0.00, 0.00]	0.00	[0.00, 0.00]	0.00	[0.00, 0.00]
Secondary school and higher	−0.47	[−1.05, 0.11]	−0.73	[−1.60, 0.14]	−0.56	[−1.87, 0.75]
Occupation						
Unemployed	0.00	[0.00, 0.00]	0.00	[0.00, 0.00]	0.00	[0.00, 0.00]
Employed	−0.02	[−1.12, 1.08]	−1.10	[−2.75, 0.55]	3.19*	[0.71, 5.67]
Support given to patient					
Finance and Care	0.00	[0.00, 0.00]	0.00	[0.00, 0.00]	0.00	[0.00, 0.00]
Finance or Care	0.46	[−0.11, 1.04]	1.14**	[0.28, 2.00]	2.25***	[0.96, 3.54]
Others	0.05	[−0.71, 0.81]	0.06	[−1.08, 1.19]	0.70	[−1.01, 2.41]
Financial difficulty					
Yes	0.00	[0.00, 0.00]	0.00	[0.00, 0.00]	0.00	[0.00, 0.00]
No	−0.80*	[−1.48, −0.12]	−0.07	[−1.08, 0.94]	−1.97*	[−3.49, −0.44]
Constant	19.34***	[17.96, 20.73]	29.86***	[27.79, 31.93]	26.19***	[23.08, 29.30]
r2	0.14		0.17		0.06
N	533.00		533.00		533.00

Note: * p < 0.05; ** p < 0.01; *** p < 0.001.

## 4. Discussion

To the best of our knowledge, this is one of the first studies about caregivers of cancer patients in Vietnam examining their uses of coping strategies and investigating the relationship between coping mechanisms and social support. Determining not only the coping styles utilized by the Vietnamese caregivers when they experience caregiver burdens but also whether there are any correlations between social supports and coping strategies is an important starting point to develop appropriate public health interventions supporting caregivers in the future.

We found that active coping, acceptance, and positive reframing were three most preferable coping strategies utilized by caregivers of patients diagnosed with cancer in Vietnam. Substance use was the least used coping mechanism of the respondents. Additionally, a significant positive correlation was found between high level of social support and mechanisms of coping.

Our findings revealed that the most utilized coping strategy among Vietnamese cancer caregivers was emotion-focused coping, while dysfunctional coping was least used.This was in accordance with reports demonstrated by other studies using Brief COPE on caregivers of cancer patients [10],[33],[41], caregivers of dependent older adult relatives [42], caregivers of stroke patients [43] or caregivers of elderly people with dementia [44]. This result indicated that Vietnamese caregivers of cancer patients tend to use positive strategies to solve their problems, which is consistent with findings from previous studies of Nguyen Thi Thanh Mai et.al. on coping strategies in Vietnamese parents of children with cancer [35],[36]. However, in our study, emotion-focused coping was preferred to use than problem-focused coping, which differed from results of some previous studies that problem-focused coping was more often utilized [22]. In addition, one of the problem-focused strategies, active coping, is the most widely utilized coping strategy of caregivers in our study. Some previous studies indicated that people who used problem-focused coping styles more frequently than others tend to experience less anxiety, depression, and hopelessness and also experience an increase in quality of life [45]–[47].

Substance use, self-blame, and behavioral disengagement were reported as harmful coping strategies by several studies. Although creating a temporary relief from the burden, it was reported that the utilization of these maladaptive coping strategies is related to psychological distress such as depression and anxiety [41],[48],[49]. Our study showed that there was a significant portion of participants which reported that they used these unhealthy coping strategies, which were similar to the findings of other authors [50],[51]. In fact, caregivers in our study as well as widely in Vietnam were mostly family members or close relatives with cancer patients, who are quite sensitive to pressures and emotional strains that are related to cancer patients' health condition. These findings suggest some possible methods to improve effective coping in Vietnamese cancer caregivers such as family or friend support interventions.

Our study results highlight the patterns of correlations that might exist between coping strategies and levels of social support. In particular, after controlling for gender, age, educational level, occupation, types of support and financial difficulty, we found that high social support showed a significant positive association with all coping mechanisms including problem-focused coping, emotion-focused coping and dysfunctional coping. Moderate social support also had a significant positive relationship with problem-focused coping and emotion-focused coping. This aligned with findings from previous studies that social support is connected to the utilization of coping styles [27],[33],[52]. Other literature also revealed that social support can inspire proactive coping [29],[30]. In the context of Vietnam, where people often live within the communities with the same cultural characteristics and strong social cohesion, social support plays an important role in encouraging cancer caregivers to use positive coping styles to deal with their stressful situations. Additionally, Hsu and Tung showed that coping strategies had mediate effect from social support to deal with difficulties and stress symptoms [53]. This could be explained by the ability of social support in reducing harmful disengagement coping mechanisms (such as denial, self-blame or substance use behaviors) and in encouraging advantageous engagement coping mechanisms (such as active coping, planning or positive reframing). This is because someone in a person's social network may always be willing to assist them in overcoming mental health challenges [27],[52].

In addition, findings from our study also demonstrated that the 45 years and older age group and those having no financial difficulty were significantly inversely correlated with problem-focused coping style. Supporting the patient in either finances or care was significantly correlated to emotion-focused coping style. Being employed and supporting the patient in finances or care were positively associated with the dysfunctional coping style. Having no financial difficulty was significantly negatively related to dysfunctional coping style. However, there was no significant difference between male and female caregivers in the utilization of any coping strategy in our study, which is dissimilar to the previous finding of Han et al. [33]. This study has several limitations that need to be considered. First, since this is a cross-sectional study, we cannot examine whether the relationships are a result of reciprocity or causality. Accordingly, further longitudinal studies with follow-up periods should be conducted in the future to overcome this limitation. Secondly, potential selection bias could have occurred due to the convenience sampling technique utilized, which could affect the generalizability of the study results. In addition, this is a hospital-based study, therefore, our findings might not be representative of all caregivers of patients with cancer in Vietnam. Further studies should be conducted to explore any possible changes in the coping styles of caregivers of patients with cancer throughout the cancer treatment journey.

## 5. Conclusion

This study demonstrated that active coping, positive reframing, and acceptance mechanisms were the three most frequently used coping mechanisms among cancer caregivers; meanwhile, substance use was the least utilized. A significant positive correlation was found between a high level of social support and the utilization of positive coping strategies. Receiving a high level of social support and utilizing positive coping strategies enables caregivers to mitigate their caregiving burden, control the situation, and enhance the caregiver's quality of life. Therefore, it is necessary to develop appropriate public health interventions manipulating social support and positive coping strategies in order to support caregivers of cancer patients dealing with their stressful situations, thereby, help to improve patient care.
